# Supplementary material for: Mutation Associated with Orange Fruit Color Increases Concentrations of β-Carotene in a Sweet Pepper Variety (Capsicum annuum L.)
Source: Foods. 2021 May 28;10(6):1225. doi: 10.3390/foods10061225 (PMC8227262; doi:10.3390/foods10061225)
Supplement: Supplementary file 1 [file foods-10-01225-s001.zip › Suplementary S1 - Primers.pdf]

Article

Mutation Associated with Orange Fruit Colour Increases Concentrations of β-carotene in a Sweet Pepper Variety (*Capsicum annuum* L.)

Sub-title: Biochemical and Molecular Characterization of Bulgarian Mutant Pepper Variety with a High-Added Value

Nasya Tomlekova [1*], Velichka Spasova-Apostolova [2], Ivelin Panchev [3], and Fatma Sarsu [4]

1 Department of Breeding, Variety Maintenance and Introduction, Maritsa Vegetable Crops Research Institute, 32 Brezovsko shosse St., 4003 Plovdiv, Bulgaria; nasia.tomlekova@gmail.com
2 Department of Breeding and Seed Production, Tobacco and Tobacco Products Institute, 4108 Markovo, Bulgaria; vilispasova-apostolova@abv.bg
3 Department of Biochemistry, Sofia University, 8 Dragan Tzankov, 1164 Sofia, Bulgaria; ipantchev@gmail.com
4 Plant Breeding and Genetics Section, Joint FAO/IAEA Division of Nuclear Techniques in Food and Agriculture, International Atomic Energy Agency, 1400 Vienna, Austria; *F.Sarsu@iaea.org*

* Nasya Tomlekova: nasia.tomlekova@gmail.com ; Tel.: +359 (0)32 951227

Primers and primer sequences used in the present study

Table S1. Primers names and sequences positioned in the sequences of β-carotene hydroxylase genes in pepper

Primers	Sequences	Authors	Tm, °C
b-CRT 1 F	CTTCACCGTACCGTACATG	unpublished	52
b-CRT 3 F	CTTATCATGTGTTGTCTCTCG	unpublished	51
b-CrtZ 4 R	CCTTCTCTTGGTCTATGGTG	unpublished	51
b-CRT 5 F	TTGCAGTCACACCATAGA	unpublished	50
b-CRT 6 R	GAAGATCACATTTGTCAG	unpublished	45
b-CRT 7 F	TTAATCACACTTGGCACCAT	unpublished	52
b-CRT 8 R	AGAGCCTAAATACAAGTCCA	unpublished	50
FW1_H2	ATGGCTGCTGAAATTTCAATCTCCGCTAGCTCC	unpublished	65
FW1_H1	GTCCACCTCCCGTACAAGTTATTACCGCC	unpublished	64
RE1_H1	GGGAATGAGGCCTTTATGGAAGAAACCATAGTC	unpublished	62
RE2_H1	AAGCCATAAGGGACACCGTTGAACTTCTCT	unpublished	63
CrtZ-B_F	CGTACCGTACATGGCTGCT	[44]	57
CrtZ-B_R	CATCTCGCCCAGTACTCCAT	[44]	55
CrtZ-C_F	GAGCTGAACGATATTTTTGCC	[43]	52
CrtZ-C_R	TAGGAACAAGCCATATGGGA	[43]	52
CrtZ-D_F	AGATGGGCGCATAGAGCACTA	[43]	57

CrtZ-D_R	ACCCCATCAAATTTGTCCGA	[43]	54
CrtZ-E_F	CGTACATGGCTGCTGAAATT	[43]	53
BCH F	ATGGCTGTTATGGCGGTTTA	[35]	54
BCH R	ATCGTTCAGCTCGAAAGGTC	[35]	55
CrtZ_R EX1	ACCTCCATTTGCCACG	[35]	51

* Primers designed by Tomlekova et al. (2009) [43], Petrov et al. (2013) [44] and Tomlekova et al. (2016) [35]
