# Supplementary material for: Mutation Associated with Orange Fruit Color Increases Concentrations of β-Carotene in a Sweet Pepper Variety (Capsicum annuum L.)
Source: Foods. 2021 May 28;10(6):1225. doi: 10.3390/foods10061225 (PMC8227262; doi:10.3390/foods10061225)
Supplement: Supplementary file 1 [file foods-10-01225-s001.zip › Supplementary S2 - Sequence.pdf]

Article

Mutation Associated with Orange Fruit Colour Increases Concentrations of β-carotene in a Sweet Pepper Variety (*Capsicum annuum* L.)

Sub-title: Biochemical and Molecular Characterization of Bulgarian Mutant Pepper Variety with a High-Added Value

Nasya Tomlekova [1*], Velichka Spasova-Apostolova [2], Ivelin Panchev [3], and Fatma Sarsu [4]

[1] Department of Breeding, Variety Maintenance and Introduction, Maritsa Vegetable Crops Research Institute, 32 Brezovsko shosse St., 4003 Plovdiv, Bulgaria; nasia.tomlekova@gmail.com

[2] Department of Breeding and Seed Production, Tobacco and Tobacco Products Institute, 4108 Markovo, Bulgaria; vilispasova-apostolova@abv.bg

[3] Department of Biochemistry, Sofia University, 8 Dragan Tzankov, 1164 Sofia, Bulgaria; ipantchev@gmail.com

[4] Plant Breeding and Genetics Section, Joint FAO/IAEA Division of Nuclear Techniques in Food and Agriculture, International Atomic Energy Agency, 1400 Vienna, Austria; *F.Sarsu@iaea.org*

* Nasya Tomlekova: nasia.tomlekova@gmail.com ; Tel.: +359 (0)32 951227

Combined sequence of the *CrtZ*$_{chr06}$ gene (beta-carotene hydroxylase gene located on chromosome 6) in pepper - Oranzheva kapia, Pazardzhishka kapia 794 and Kurtovska kapia 1 (combined sequence of multiple sequenced clones in the 3 genotypes studied).

GTCCACCTCCCGTACAAGTTATTACCGCCATAGCCCATTTCTCGGCCCAAAACCCACTCC
AACAACCCCATCAGTGTACCCCATAACTCCGTTTTCTCCTAATCTCGGCTCAATTCTGAG
GTGTAGAAGGAGACCCAGCTTCACTGTTTGCTTTGTTCTTGAGGATGACAAGTTCAAAAC
CCAATTTGAGGCTGGGGAAGAAGATATTGAAATGAAGATTGAAGAGCAGATCTCAGCGAC
GCGTTTGGCGGAGAAATTGGCTAGGAAGAAATCGGAGAGGTTTACTTATCTTGTTGCTGC
GGTTATGTCTAGTTTTGGGATTACTTCCATGGCTGTTATGGCGGTTTATTACAGATTTTA
CTGGCAAATGGAGGTACTGGAACATTCCATAATCCCCAAACTCTTCTTCTTTTGGGACTT
AAATTTTATTGTAATTTTATTTCTGTTTCGAGTGTAGGGTGGAGAAGTTCCTTTTTCCGA
AATGTTTGGCACATTTGCTCTCTCTGTTGGTGCTGCTGTAAGTTCCATTTCTGTCTTATT
ATGAGCTAGTAATTGTAAGATTAGATACTGGCCTAATTCATACCCCCAAAAGAGCTAGCC
GATGAGGAGAATATTGTTCAAGTCCGACCCATATCATTAACCATCAATATGGACTTTTTC
ATTAATAAATTAGTCGGCAAAAATATTGTAACGGTTGAAAAGTATTATTCCGTAATTACT
GGAGAAACATTAATAAGTCGAAATTCAAATTACAAGTGGTTTAAAAGTTTGAAATTTTCA
AAAATATTTTTGAATCATTCTAGAATAGTTAACAAGAGAGTAACTAGAAATAGCAGAGTT
GGTTCTTCGGTGATCGATTTTAAAATGTCTGATGAAAATTGAATTGGAATTTCAGGTAGG
AATGGAGTTTTGGGCTAGGTGGGCACATAAAGCGCTATGGCACGCCTCTTTATGGCACAT
GCACGAGGTTTGTGTGCTACTTATATGGATAGTAATTAAATGCCTTATTTCATTCTTGAT
GAGAAAAGAATTACTTAAGATAATTTAGTACGTACTTTATATTAAGATTACTGTCGTTAC
ATTAAATTGTTGCAGTCACACCACAAACCAAGAGAAGGACCTTTCGAGCTGAACGATGTT

TTTGCCATAATCAATGCTGTTCCAGCTATAGCCCTCCTTGACTATGGTTTCTTCCATAAA
GGCCTCATTCCCGGCCTCTGCTTCGGCGCTGTATGTCATCATTTGGCTTGCACTTTTTTT
TAAAAAATAATGTTAAAGTATGATCTTGATGGGGCACAAATTCAAAATTTAAAATAAGAT
AATCCATAATGGACGTAATCTATATTTATAAACTTCAAAATTCTTTTTTCATATAATGTA
AATGAATTGAAAGCAGTTAGATAGCTAAACATGTTGATCTTGCTCTAAGTCCCCCTCTAA
TATTTCCCTAGGATACTCCAGAAATTTTACTCGATTAAGACGCTGTCTATTGCTTAGTGT
AAATTAATGAGTATAGTTGTAAATATTTTATTTACATCAAGTCTAAACTCGTTTTAAGTT
CAACGTTAAATTGATGAAGCGGTCTTCTGTTATGCAGGGGTTAGGGATCACAGTATTTGG
GATGGCATACATGTTCGTTCATGATGGTTTGGTTCACAAGAGATTCCCTGTTGGACCTGT
AGCTAATGTACCTTATCTTAGGAAGGTGGCTGCAGCTCATTCGGTAATTTAATCACACTT
GAATGCGATTTTTATGAAGTACTATTAATTGAAATTTGTTCATAAGATTTTCTAATGTAT
TGGTTTGCTACTGCATTTGCAGCTTCATCATTCAGAGAAGTTCAACGGTGTCCCTTATGG
CTT
